# Improving adolescent sexual and reproductive health in Latin America: reflections from an International Congress

**DOI:** 10.1186/1742-4755-12-11

**Published:** 2015-01-24

**Authors:** Kathya Córdova Pozo, Venkatraman Chandra-Mouli, Peter Decat, Erica Nelson, Sara De Meyer, Lina Jaruseviciene, Bernardo Vega, Zoyla Segura, Nancy Auquilla, Arnold Hagens, Dirk Van Braeckel, Kristien Michielsen

**Affiliations:** South Group, C. Ecuador O-138, Edificio Holanda, A-3A, Cochabamba, Bolivia; Reproductive Health and Research, World Health Organization, 20 Avenue Appia, CH - 1211 Geneva 27, Switzerland; International Centre for Reproductive Health (ICRH), Ghent University, De Pintelaan 185 6 K4, 9000 Gent, Belgium; Center for Social Science and Global Health, University of Amsterdam, Nieuwe Achtergracht 166, 1018 WV Amsterdam, The Netherlands; Department of Family Medicine, Lithuanian University of Health Sciences, Kaunas, 44307 Lithuania; University of Cuenca- Facultad de Ciencias Médicas, Avenida 12 de abril S/N sector El Paraíso, Cuenca, Ecuador; Instituto Centroamericano de la Salud, Reparto Los Robles, Restaurante La Marsellaise 1 c. al norte 1 c. al este, casa # 77, Managua, Nicaragua

**Keywords:** Adolescents, Latin America, Sexual and reproductive health, Policy, Intervention strategies, Teenage pregnancies

## Abstract

In February 2014, an international congress on Promoting Adolescent Sexual and Reproductive Health (ASRH) took place in Cuenca, Ecuador. Its objective was to share evidence on effective ASRH intervention projects and programs in Latin America, and to link this evidence to ASRH policy and program development. Over 800 people participated in the three-day event and sixty-six presentations were presented.

This paper summarizes the key points of the Congress and of the Community Embedded Reproductive Health Care for Adolescents (CERCA) project. It aims at guiding future ASRH research and policy in Latin America.

1. Context matters. Individual behaviors are strongly influenced by the social context in which they occur, through determinants at the individual, relational, family, community and societal levels. Gender norms/attitudes and ease of communication are two key determinants.

2. Innovative action. There is limited and patchy evidence of effective approaches to reach adolescents with the health interventions they need at scale. Yet, there exist several promising and innovative examples of providing comprehensive sexuality education through conventional approaches and using new media, improving access to health services, and reaching adolescents as well as families and community members using community-based interventions were presented at the Congress.

3. Better measurement. Evaluation designs and indicators chosen to measure the effect and impact of interventions are not always sensitive to subtle and incremental changes. This can create a gap between measured effectiveness and the impact perceived by the targeted populations.

Thus, one conclusion is that we need more evidence to better determine the factors impeding progress in ASRH in Latin American, to innovate and respond flexibly to changing social dynamics and cultural practices, and to better measure the impact of existing intervention strategies. Yet, this Congress offered a starting point from which to build a multi-agency and multi-country effort to generate specific evidence on ASRH with the aim of guiding policy and program decision-making. In a region that contains substantial barriers of access to ASRH education and services, and some of the highest adolescent pregnancy rates in the world, the participants agreed that there is no time to lose.

## Resumen

En febrero de 2014, un congreso internacional sobre la promoción de la Salud Sexual y Reproductiva de los adolescentes (SSRA) tuvo lugar en Cuenca, Ecuador. Su objetivo era compartir evidencia sobre proyectos y programas de intervención eficaz en SSRA en América Latina, y vincular esta evidencia a la política de SSRA y al desarrollo de programas. Más de 800 personas participaron en un evento de tres días con sesenta y seis presentaciones.

Este documento resume los puntos clave del congreso y del proyecto CERCA (Cuidado de la Salud sexual y Reproductiva para Adolescentes enmarcada en la comunidad). Su objetivo es orientar la investigación futura y la política de SSRA en América Latina.

1. El contexto es importante. Los comportamientos individuales están fuertemente influenciados por el contexto social en el que se producen a través de factores determinantes a nivel individual, relacional, familiar, comunitario y social. Las normas de género, actitudes y facilidad de comunicación son dos factores determinantes.

2. Acción innovadora. Hay evidencia limitada e irregular de los enfoques eficaces para llegar a los adolescentes con intervenciones que necesitan llevarse a escala. Sin embargo, existen varios ejemplos que fueron presentados en el congreso y que se ven prometedores e innovadores porque proporcionan una educación integral de la sexualidad a través de los métodos convencionales y el uso de nuevos medios de comunicación, la mejora del acceso a servicios de salud, y uso de intervenciones comunitarias para llegar a los adolescentes, familias y su comunidad.

3. Mejor medición. Los diseños de evaluación y los indicadores elegidos para medir el efecto e impacto de las intervenciones no siempre son sensibles a los cambios sutiles y graduales. Esto puede crear una brecha entre la eficacia de medidas y el impacto percibido por las poblaciones beneficiarias.

Por lo tanto, una conclusión es que necesitamos más pruebas para determinar mejor los factores que obstaculizan el progreso en la SSRA de América Latina, para innovar y responder con flexibilidad a los cambios en la dinámica social y las prácticas culturales, y para medir mejor el impacto de las estrategias de intervención existentes. No obstante, este congreso proporcionó un punto de partida para construir un esfuerzo común entre las multi-agencias y multi-país que genere evidencia específica sobre SSRA con el objetivo de orientar la toma de decisiones políticas y programas. En una región que aún tiene barreras de acceso a la educación y los servicios de SSRA y se encuentran las tasas de embarazo adolescente más altas del mundo, los participantes coincidieron en que no hay tiempo que perder.

## Background

### The International Congress on promoting adolescent sexual and reproductive health

In February 2014, an International Congress on Promoting Adolescent Sexual and Reproductive Health (ASRH) took place in Cuenca, Ecuador. This Congress was the culmination of a four-year, multi-country intervention-research study titled the “Community Embedded Reproductive Health Care for Adolescents in Latin America” (CERCA) project [1]. CERCA was funded by the European Commission’s 7th Framework Program. The Study endeavored to develop and test a package of ASRH interventions that could be delivered in collaboration with existing public health systems and in conjunction with community actors. The project was implemented in three Latin American cities: Managua, Nicaragua; Cochabamba, Bolivia and Cuenca, Ecuador.

As a research project, CERCA both generated new evidence on social determinants of ASRH, and developed innovative strategies for promoting ASRH at the community level with ongoing participation and inputs from stakeholders such as adolescents, parents and adult family members of adolescents, educators and health professionals. Controlled intervention trials have been conducted in the three cities. Interventions were implemented from August 2011 to April 2013 in randomly chosen town districts in Managua and in purposively selected secondary schools in Cochabamba and Cuenca. In order to assess the impact of the interventions we compared the change in reports of selected behaviours between adolescents from intervention groups and control groups. The total number of respondents that participated in both the baseline and the post-intervention survey was 2,642.

The objective of the Congress was to present innovations and share lessons learned in the design and delivery of clinical and educational interventions – with a focus on outreach- ASRH actions that are evidence-based, and to link this evidence to ASRH policy development. Over three days, sixty-six presentations were given and over 800 people participated. The congress resulted in the ‘Cuenca Declaration on ASRH in Latin America’ which stresses the need for meeting the needs and fulfilling adolescents’ rights to reliable sexuality education and to good quality sexual health services in Latin America [2].

This paper combines key lessons learned from the CERCA project and key issues raised in the Congress that are relevant for guiding ASRH research and policy in Latin America. It is the result of consultations with the main CERCA partners and Congress organizers. In a region that has barriers of access to ASRH education, services and some of the highest adolescent pregnancy rates in the world there is no time to lose.

### Adolescent sexual and reproductive health in Latin America

The point of departure the CERCA project and the Cuenca Congress was the observation that adolescents in Latin America continue to face serious SRH problems and substantial barriers to SRH education and services. Regional data shows that the majority of sexually active adolescents do not consistently use modern contraceptive methods to prevent pregnancy or sexually-transmitted infections (STIs) [3]. Of the estimated 1.2 million unplanned pregnancies in the region, half occur during adolescence [4]. Up to 50% of the women in the region give birth for the first time during their adolescence. Teenage pregnancies are associated with a higher incidence of maternal complications during pregnancy and delivery, especially for younger adolescents [5, 6]. Children of adolescent mothers are also at increased risk of neonatal mortality, preterm birth and low birth weight [7, 8]. Given that abortion remains highly restricted in all three CERCA countries, and in most other countries in Latin America, there are limited options for a young person faced with an unwanted or unplanned pregnancy [9]. In the face of this public health crisis, quality evidence can help Latin American governments develop and implement sound policies and programs.

### Key lessons

#### Context matters

Individual behaviors are strongly influenced by the social context in which they occur, through determinants at the individual, relational, family, community and societal levels. Gender norms/attitudes and ease of communication on ASRH are two key determinants.

Early pregnancy and poor reproductive outcomes among adolescents are determined by a web of micro-, meso- and macro-level factors. Individual choices to engage in specific behaviours are shaped by social, economic and cultural factors that operate at the individual, interpersonal (couple, peer group), family and community level. This ecological approach can help identify the determinants in ASRH, and can be used to develop better strategies, and ways to monitor and evaluate them. The CERCA project and the Congress contributed to additional insights into determinants of ASRH. The CERCA project demonstrated that positive gender attitudes are of critical importance. Personal attitudes towards gender equality appeared to have a high predictive value for adolescents’ sexual behaviour and experiences and partner communication. More egalitarian gender attitudes are related to higher rates of contraceptives use within the couple, more positive experiences of sexual intercourse and better communication about sex with the partner among sexually active and sexually non-active adolescents [10, 11]. As the prevailing norms link women’s status to their fertility, a significant proportion of teenage pregnancies are, in fact, wanted. Borile’s study [12] suggests that the desire to become a teenage mother is related to gendered role patterns, social recognition, cultural factors, and limited economic and professional opportunities [13]. These views were reinforced by anecdotal evidence and professional experiences presented at the conference. For example gender norms influence young men’s decisions not to use contraception since “a macho” is expected have children everywhere [14, 15].

The CERCA project’s findings further highlight the importance of communication. Firstly, feeling comfortable to talk about sexuality with friends is positively associated with condom use. Boys and young men who find it easy to talk with their partner about sexuality issues report a greater likelihood of hormonal contraceptives use by their partners [16, 17]. Secondly, ethnographic data collected through participatory research processes and peer group discussions revealed much room for improvement in adult-child communication on sexuality which is often characterized by silence, implied expectations and gendered conflicts [18]. In order to have a substantial effect, interventions need to take into account this multitude of determinants at the levels of both their development and implementation.

#### Innovative action

There is limited and discontinuous evidence of effective approaches to reach adolescents with the health interventions they need at scale. Yet, there exist several promising and innovative examples of providing comprehensive sexuality education through conventional approaches and using new media, improving access to health services, and reaching adolescents as well as families and community members using community-based interventions.

Here is a description of some successful intervention experiences which involve multidisciplinary approaches and ways to measure.

### Comprehensive sexuality education

The congress emphasized that children and young people are rightfully entitled to age and developmentally appropriate and correct information on SRH. One issue that often rose in the Congress was that public educators and health centers provide limited sexuality education that focuses on risk-reduction and negative messages. During the CERCA project, country partners asked local populations of adolescents^a^ what topics they would like to see included in sexuality education. In Bolivia, this resulted in an education strategy that included personal and intimate partner communication techniques, self-esteem building, positive gender attitudes and gender equality, a life project, conflict management, and available health services [19]. Many Congress presentations included a plea for comprehensive and developmentally appropriate sexuality education as the bedrock for attitude formation and decision-making [20, 21].

### Adolescents’ access to health services

One of CERCA project’s central objectives was to increase access to health services for adolescents. One of the main barriers to adolescents access to contraception is that they do not trust the stated confidentiality of health facility staff and are worried about being judged negatively for being sexually active [22]. This is particularly the case for young girls. Furthermore, health workers feel inadequately equipped to deal with adolescents seeking contraceptive counseling: they are confused about legal codes, parental consent and moral concerns, as well as unable to cope with practical constraints such as limited personnel and opening hours [23, 24]. A qualitative study^b^ done by Nelson demonstrated that, according to health service providers, adolescents grow up in an environment where sexuality is a taboo issue hindering their sharing information on sexual health at the familial and educational levels. Health service providers blame ‘the culture of taboo’ as a barrier to access to ASRH services, however, they do not seem to be aware that their own attitudes and reactions regarding to adolescents, in particular girls, who ask information and services related to ASRH have a strong negative impact as well [25]. Congress participants concluded that any SRH promotion strategy must necessarily pay attention to health service providers’ attitudes as well as wider social and cultural norms in addition to health infrastructure and contraceptive supply chains [26, 27].

### Using text messages to reach adolescents

Mobile phone, smart phone, and internet and social media use is on the rise among young people in Latin America. Borile and Cordova Pozo [28] stressed that the changing technological and communication landscape has opened new opportunities for sexuality education, sexual health promotion and advocacy efforts in the region. New media offers a valuable tool for recruiting and mobilizing adolescents to use already-existing public health services and to act as ‘first responders’ to the questions and doubts that can create barriers to health service access.

As part of the CERCA project in Bolivia, adolescent-friendly text messages were used for cost-effective and efficient adolescent outreach, resulting in an overwhelming response from adolescents. Over a period of 18 months, 507 questions on ASRH issues were received by text messages on a bidirectional text-messaging base linking CERCA-and adolescents. An evaluation with adolescents revealed that receiving a text message with health advice and having the opportunity to ask questions reduced the obstacles to those who normally would not access health centers due to stigma, taboo, costs or long waiting times [29]. The large number of questions and different sensitive topics broached by adolescents in a short time showed that text messages have the potential to break down the barriers between the health center/health service professional and the adolescent and can motivate adolescents to seek help [30]. While this is not sufficient on its own, such an approach should be embedded in a broader context that includes addressing providers’ attitudes, it can contribute to the beginnings of change in adolescents’ attitudes and the initiation of dialogue on difficult topics.

### Community-based interventions

Given the important influence of determinants at the meso- and macro-level on individual behavior, it seems evident that ASRH promotion interventions should address the wider community. However, this is often not yet the case. In her presentation, Segura explained that in Managua, due to the relatively high levels of young people out-of-school in the CERCA project’s selected neighborhoods, the local consortium partner chose to carry out intervention activities at the neighborhood level (e.g. mobile cinemas, sporting events, door-to-door outreach and education campaigns). Friends of Youth (FoY) were the driving forces of the community interventions. FoY are young adults, intensively trained in ASRH. They served as mentors for adolescents in their community and helped them build their competence to make deliberate choices. In addition, they referred them to appropriate health service providers when needed. Besides one-to-one interactions with adolescents, the FoY also supported community activities including workshops, exhibitions, street theatre and awareness campaigns. Another action that was used in this type of setting was the “Movisex”, an adapted car aimed at reaching adolescents who are out of school, providing face-to-face education and information. The evaluation of these activities was done through in-depth interviews by Nelson who concluded that these community-oriented interventions were highly appreciated by the community.

### Adult and family involvement

During the CERCA project young people indicated that they wanted to learn more from adults about how to negotiate within a romantic relationship about contraceptive use, how to prepare for decision-making related to sex, and how to deal with tensions in communication with adult family members [18]. Several Congress presentations indicated that the involvement of the family in ASRH interventions is important: family can create or destroy an environment to address ASRH [20, 21, 31].

#### Better measurement

Evaluation designs and indicators chosen to measure the effect and impact of interventions are not always sensitive to the subtle and incremental changes that occur. This can create a gap between measured effectiveness and the impact of interventions perceived by the targeted populations.

While the qualitative effectiveness evaluation of CERCA did demonstrate some positive effects -on condom use and overall knowledge and use of sexual health services in Ecuador, on ease of communication in Bolivia, but these results were not quantitative measurable. Ethnographic research suggested that the chosen quantitative indicators and measurements used in the Project did not capture the complexity of social determinants of ASRH or the shifting gender and power dynamics at the family and community level that influenced the ways in which intervention activities were received and acted upon. Quantitative measures only could focus on one or two aspects of the intervention, neglecting the multidimensional approach that the interventions took [18]. Furthermore, the limited evidence regarding the effectiveness of the interventions may be due to the fact that randomized controlled trials alone are not capable of capturing the full complexity of a community-embedded intervention process. Or maybe because of the lesser resources given for process evaluation and rigorous qualitative research and the lesser status given to ‘soft’ evidence within the context of public health interventions.

Although little quantifiable positive impact of the CERCA interventions could be measured, different presentations indicated that improvements were made possible by the CERCA project. Bersosa J., from Ecuador [24], presented the effects at the local level, where adolescent SRH networks were established with city government funding, health centers were made adolescent-friendly and sexuality education in schools broadened their horizons. There were clear impacts at the policy level too as was highlighted in the presentations from Malo M. and Guijarro S. (Ministry of Health in Ecuador) [25, 32, 33] who talked about the contribution of the Project to the development of national strategies for adolescent pregnancy prevention and creating a vision for improving policies in ASRH and health services.

Complex problems require comprehensive solutions and tailored evaluation designs. This can seem at odds with the increasing call for evidence-based policy making and programme development. While we continue to develop and test effective approaches to provide adolescents with the most effective sexuality education and SRH services, we must use the available evidence to respond to the needs of adolescents today and to fulfill their right to sexuality education and health. Process evaluations are a crucial part of this. What constitutes evidence needs to be reflected upon further among programme implementers, evaluation researchers and policy makers.

## Conclusions

Research presented at the congress reiterated that individually-targeted ASRH interventions are not sufficient to bring about change in adolescents’ sexual behaviours, but rather that extended family networks, communities, local and regional actors must also be involved. This idea corresponds to the Andean Plan to Prevent Teen Pregnancy (PLANEA) an initiative of the Ministries of Health of Bolivia, Chile, Colombia, Ecuador, Peru and Venezuela, that indicates that establishing strong bonds between young people and their teachers, health professionals, parents, and friends can contribute to effective programmes. When teenagers develop strong relationships in a secure, safe environment, they can build the life skills they need to take control of their own destinies. Alongside this focus on the individual, relational and community levels, we agree with the conclusions of the International Interagency Meeting on Current Evidence, Lessons Learned and Best Practices in Adolescent Pregnancy Prevention in Latin America and the Caribbean that there should also be a clear focus on the macro-level with a political and financial commitment to create empowering legal frameworks and to implement sexual and reproductive health programmes for adolescents.

Better mechanisms and measurements are needed to produce the kind of evidence that can guide policy and programme development reflecting the complex nature of adolescent sexuality and sexual behaviors. It is too easy to look at the ‘problem’ of adolescent pregnancy through one disciplinary lens, be it medical, legal, economic or political. In order to develop the necessary political and programmatic frameworks to shift ASRH in a positive direction, multi-disciplinary and comprehensive approaches are necessary. This Congress provided a starting point to building a multi-agency, multi-sector effort to generate improved evidence and strategies which could contribute to reducing gaps in access to healthcare services in an equitable manner, taking into account cultural issues and social participation. In a region that contains pockets of stagnant modern contraceptive use, substantial barriers of access to ASRH education and services and some of the highest adolescent pregnancy rates in the world, there is no time to lose.

### Endnotes

^a^This data was collected in the pre-intervention quantitative survey as well as through pre-intervention and intervention period qualitative research consisting of peer discussion groups, focus discussion groups, in-depth interviews, and participatory ethnographic research.

^b^This qualitative study included in-depth interviews to adolescents and health personnel that participated in CERCA research.

## Authors’ information

^1^Researcher and project leader at South Group, ^2^Scientist, Adolescent Sexual and Reproductive Health, World Health Organization, ^3^Researcher & CERCA team leader at ICRH, ^4^Post-doctoral Research Fellow, University of Amsterdam AISSR and Medical Anthropology Departments, ^5^Researcher at ICRH, ^6^Professor associated at Kaunas University of Medicine, ^7^CERCA Project Director in Ecuador, ^8^Researcher at ICAS, ^9^Congress coordinator, ^10^Researcher at South Group,^11^Financial-administrative director at ICRH, ^12^PhD in Social Medical Sciences on adolescent sexual and reproductive health.

## Abbreviations

ASRH: Adolescent sexual and reproductive health
CERCA: Community Embedded Reproductive Health Care for Adolescents
FoY: Friends of Youth
IUD: Intrauterine device
LARC: Long acting reversible contraception
NGO: Non-governmental organizations
SASIA: Sociedad Argentina para la Salud Integral del Adolescente
SRH: Sexual and reproductive health
STI: Sexually transmitted infections.
